# Case report: Complex left-carina resection: three-year single-center experience

**DOI:** 10.3389/fonc.2024.1367311

**Published:** 2024-03-18

**Authors:** Simone Tombelli, Domenico Viggiano, Ottavia Salimbene, Marco Trigiani, Luca Voltolini, Alessandro Gonfiotti

**Affiliations:** ^1^ Division of Thoracic Surgery, Careggi University Hospital, Florence, Italy; ^2^ Division of Interventional Pulmonology, Careggi University Hospital, Florence, Italy

**Keywords:** tracheal sleeve pneumonectomy, carina, carinal pneumonectomy, tracheobronchial angle, complex tracheobronchial resection

## Abstract

Carinal and tracheobronchial angle tumors have long been a contraindication for surgical removal; the technique of tracheal sleeve pneumonectomy makes it possible to approach this malignancy but still represents a surgical challenge. Left sleeve pneumonectomy is less common compared with right sleeve pneumonectomy and represents a minority component in the literature’s case series due to the complexity of the anatomy. In addition, there is no standard for treatment strategy, and it must be assessed on a case-by-case basis. From 2020 to 2023, we performed three left tracheal sleeve pneumonectomies and one neocarina reconstruction surgery for benign lesions without lung resections. All cases were performed without cardiovascular support such as cardiopulmonary bypass and via median sternotomy. With a median length of stay of 21.5 days (between 14 days and 40 days), all patients were transferred to a physiotherapeutic rehabilitation facility for functional reactivation, where they received physiotherapeutic respiratory therapy given the slow functional recovery. The recorded 30-day mortality was 0. There is no standardized approach for left-sided sleeve pneumonectomy, and it is still a surgical challenge due to intraoperative and postoperative difficulties.

## 1 Introduction

For a long time, tumors arising less than 2 cm from the carina or with carinal invasion were considered inoperable and were treated with chemoradiotherapy (1), but, with the improvement of surgical techniques and advances in anesthesia, these malignant tumors can be resected by tracheal sleeve pneumonectomy (TSP). The first right pneumonectomy procedure with lateral resection of a tracheal wall was described by Abbott in 1950 (2); in 1959, Gibbon published the first TSP (3). It took until 1972 for Jensik to publish a consistent series of 17 patients who underwent this procedure, which was then updated in 1982 by the same authors in a series of 34 patients (4).

Over the years, several authors reported their personal experiences with encouraging long-term oncologic outcomes (5, 6). However, resection of tracheo-bronchial bifurcation (with or without pneumonectomy) is still considered a surgical challenge in terms of intraoperative and postoperative management, and there is no standardized approach.

## 2 Report

From 2020 to 2023, we performed three left tracheal sleeve pneumonectomies and one neocarina reconstruction surgery for benign lesions without lung resections (**Table 1**). All cases were performed without cardiovascular support such as cardiopulmonary bypass (CPB) and via median sternotomy.

**Table 1:** Patient’s list.

	Gender	Age	Comorbidities	Intervention	Surgical access	Operative time (min)	Ventilation	ICU (h)	LOS (days)	Adverse event	Histology	Survival
**Patient #1**	Male	55	Carinal trachea-esophageal fistula, jejunostomy, and gastrostomy	Neocarina	Median sternotomy	210	Cross-field ventilation	48	14	None	Tracheo-esophageal fistula	Alive (3 years after surgery)
**Patient #2**	Male	44	Pulmonary mass, active smoker, and blood hypertension	Left TSP	Median sternotomy	210	Cross-field ventilation	72	22	None	NSCLC	Death 18 months after surgery (systemic recurrence)
**Patient #3**	Male	67	Pulmonary mass, active smoker, diabetes mellitus, previous acute myocardial infarction, and previous pancreatitis	Left TSP	Median sternotomy + posterolateral thoracotomy	360	Apneic oxygenation	72	15	None	Synovial Sarcoma	Death 18 months after surgery (systemic recurrence)
**Patient #4**	Female	72	Pulmonary mass, obesity, diabetes mellitus, and blood hypertension	Left TSP	Median sternotomy	180	Apneic oxygenation	960	40	Gastric paralysis and vocal cord mobility deficit (inhalation)	NSCLC	Alive (3 months from surgery)

The first case discussed is a 55-year-old male patient with a benign carinal tracheo-esophageal fistula. Because of the injury, the patient was fed via a jejunostomy and had an aspiration gastrostomy. The case was investigated with a CT of the chest and later with bronchoscopy to confirm the presence of a tracheoesophageal fistula. After discussion in the multidisciplinary meeting, the surgical indication was made, and, thus, the repair of the fistula with the creation of a neoacarina was indicated. In this case, the airway was approached through a median sternotomy with the intention of suturing the right main bronchus to the left in order to create the neocarina with the most anatomical reconstructions. The creation of the neobifurcation was performed as the anatomical conditions with the surrounding structures allowed; this represents a possible limitation to the possibility of performing this type of surgical reconstruction and, therefore, limits the number of cases. As taught by Grillo (7), a laryngeal release does not translate to relaxation at the carina. After the sternotomy, the pericardium was opened, and, then, a rigid retractor was placed between the superior vena cava and the aortic arch to allow visualization of the carina. The trachea was then mobilized in its distal part and the first 2 cm of the two main bronchi to preserve the greatest possible blood supply. The anterior esophageal wall was then repaired with a suture of the anterior muscular part after the anesthesiologist had placed a nasogastric tube. Once the airway was mobilized, a cold blade resection was performed at the level of the left and right main bronchus downstream of the lesion (this is not the case for a resection of a tumor lesion, and the margins were not explored intraoperatively) and at tracheal level upstream.

**Figure 1:**
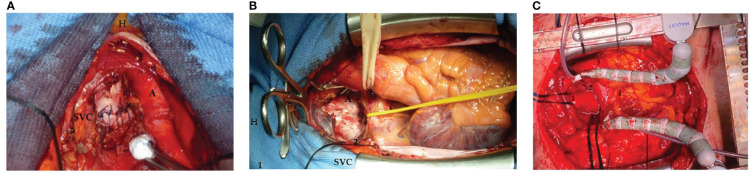
**(A)** Neocarina packed by approximating the medial walls of the right and left main bronchi to each other to form a new carina with the trachea with a continuous polypropylene suture (Prolene, Ethicon, Sommerville, NJ): superior vena cava (SVC), aorta (A), and patient’s head (H). **(B)** A traditional retractor (1) placed between the superior vena cava (SVC) and aorta **(A)** to expose the carina (2) [patient’s head (H)]. **(C)** The carina exposed trough a median sternotomy: 1, trachea; 2, left main bronchus; 3, right main bronchus; 4, heart; and 5, descendent aorta.

The neocarina was then packed by approximating the medial walls of the right and left main bronchi to each other to form a new carina with the trachea, as the mobility of the left main bronchus is restricted by its relations to the aortic arch. The two edges are then sutured together with a continuous polypropylene suture (Prolene, Ethicon, Sommerville, NJ). During this time, the lungs were alternately ventilated with apneic intermittent oxygenation. Suturing the two bronchi together tethers the newly created carina to the level of the lower edge of the aortic arch. Up to this level, the trachea was mobilized by pulling it downward to allow completion of the anastomosis, which was performed with continuous suturing using the same suture described above (**Figure 1A**). Once the anastomosis was completed, ventilation was restored via the extra-long monolumen orotracheal tube, and the suture leakage test was performed.

The other three cases were left tracheal sleeve pneumonectomies programmed for malignant lesions: two male patients and one female patient with a mean age of 61 years (range, 44–72). Two patients were active smokers at the time of surgery and two suffered from type 2 diabetes mellitus under oral antidiabetic therapy. All cases were discussed in a multidisciplinary team, and preoperative chest CT, PET-CT, and bronchoscopic examination were performed to identify airway wall involvement and determine the type of surgery. All patients underwent blood gas tests and respiratory function tests that were compatible with the planned surgery.

All three cases were operated on via a median sternotomy. The pericardium was then opened in a cranio-caudal L-shape, and, after opening the two pleural spaces, the left anonymous vein, the superior vena cava, and the ascending aorta were isolated (individually and then together with the right pulmonary boot). In the first case, we use a traditional retractor placed between the superior vena cava and aorta to expose the carina (**Figure 1B**). However, in addition to a spatial burden, this also posed an increased risk of injury to the vessel walls related to the rigidity of the instrument itself. It was then decided to use an octopus-type retractor that allows the vascular structures to be mobilized and thus expose the carina while causing less trauma to the walls of the great vessels (**Figure 1C**). This reduces the risk of damage to the vessels and creates a smaller space footprint being able to direct the body of the retractor where the surgeon wants it most.

We then isolate the two main bronchi and the trachea with a band. The lymphadenectomy of station 7 should be performed at this moment: dissection of the left vascular hilum, sequential isolation of the left upper vein and the main artery, and subsequent dissection with mechanical suturing. Once it is confirmed that resection and reconstruction are possible, we proceed with resection of the carina, cold blade section of the trachea and right main bronchus.

In the first case of TSP described, a monolumen tube was used for contralateral lung ventilation after airway resection, which was inserted from the surgical field directly into the right main bronchus and connected to the ventilator to continue ventilation. However, this technique requires the presence of the tube in the surgical field and thus limits the surgeon’s view and available space. In our experience, we have, therefore, switched to the apneic oxygenation technique. After completion of the resection, hyperoxygenation is initiated by placing a small catheter (10F) across the surgical field into the contralateral main bronchus and connecting it to a sterile line that continuously delivers 10–15 l/min O_2_ under minimal breathing pressure (0–1 mmH). This significantly reduces the size of the occupied surgical field. We continue with the end-to-end anastomosis between the trachea and the right main bronchus with a continuous polypropylene sutures (Prolene, Ethicon, Sommerville, NJ). Negative suture tightness test for air leakage is mandatory. After reconstruction, patients are ventilated with controlled pressure through the original tube (**Supplementary Videos 1**, **2**).

In one case, it was recorded the presence of persistent adhesions between the left lower lobe, the site of the known tumor that completely occupies it, and the thoracic wall with doubtful infiltration of the VI coast. It was, therefore, not possible to free the posterior parenchyma, so it was decided to perform video-assisted thoracic surgery (VATS) in the right lateral decubitus shortly after closure of the sternotomy in a fashion standard; anterior thoracotomy according to the standard plan, from which the thoracoscope was inserted; partial dissection of the posterior adhesions, revealing infiltration of the wall requiring rib resection, for which a posterior thoracotomy with removal of the posterior arch of the sixth rib is performed under thoracoscopic view; and complete mobilization of the lung parenchyma and subsequent extraction en-bloc with the part of the resected coast. During the closure of the anterior surgical access, massive bleeding occurred, so it was necessary to pack the posterolateral thoracotomy, which was performed by connecting the two previous surgical accesses. It shows the origin of the haemorrhage from the suture of the superior lobar vein, which was repaired with continuous polypropylene sutures (Prolene, Ethicon, Sommerville, NJ).

All four patients described were transferred to the intensive care unit (ICU) monitoring after surgery. In the first three patients described, the average length of stay was 64 h (range, 48–72 h), during which they remained hemodynamically stable without pharmacological support. The female patient who underwent a left TSP was transferred to the ICU at the end of the operation for postoperative monitoring. Coronarography was performed 24 h after surgery due to an increase in myocardial troponins and suspected hypokinesia on control echocardiography: No coronary disease was found. Because of the progressive anemia, it was necessary to perform blood transfusions. Back on the ward, there was an episode of ab ingestis with loss of consciousness, so the patient was intubated with an orotracheal tube, sedated, and transferred back to the ICU for the necessary treatment.

Initially, the patient was mechanically ventilated via the orotracheal tube. On the fourth day after the operation, the patient was exubated and switched to high-flow oxygen therapy alternating with non-invasive ventilation cycles. In a septic state with respiratory failure, she was orotracheally intubated again 5 days after eustubation, and, 2 days later, a surgical tracheostomy was packed. Throughout the clinical course, she was frequently recruited and bronchoaspirated to detect secretions in the setting of ineffective cough and poor expectoration.

The course of all patients was characterized by a slow recovery of motor activity with gradual weaning from oxygen therapy until its complete removal. Fibrobronchoscopy tests were performed during hospitalization, which showed good suture tightness without air leakage and good tissue trophism.

With a median length of stay of 21.5 days (between 14 days and 40 days), all patients were transferred to a physiotherapeutic rehabilitation facility for functional reactivation, where they received physiotherapeutic respiratory therapy given the slow functional recovery.

The patient who was treated with a neocarina is still alive 3 years after surgery and has no comorbidities. Two patients died 17 months and 18 months after surgery with disease recurrence and systemic metastases.

One patient, the most recent one, is still alive 3 months after surgery. At discharge to the rehabilitation and respiratory facility, which occurred 40 days after surgery, the patient shows a gradual and progressive recovery; apiretic, no antibiotic therapy; negative infection indices in blood tests; spontaneous breathing through tracheostomy; and good respiratory exchange on blood gas analysis.

## 3 Discussion

TSP is now the recommended treatment for non–small-cell lung cancer (NSCLC) invading the main bronchus, arising less than 2 cm distal to the carina (8) or involving the tracheobronchial angle with an extension of no more than three cartilage rings (9), and for other low-grade malignancies as well as benign disease involving the carina. However, due to the technical complexity, this procedure is only performed by a few highly qualified centers worldwide. Despite the high morbidity and mortality characteristic of this reconstructive technique of the tracheobronchial tree (from 11% to 53% and from 4% to 41%, respectively) (4, 6, 10, 11), recent series show good results (10–14) thanks to the improvement of the surgical technique and the intra- and post-operative management, making this surgery safe and acceptable in terms of long-term outcomes.

Among tracheal sleeve pneumonectomies, there is a notable difference between right and left pneumonectomy. The left is by far the rarest, as access to the carina trachea is restricted, leading to more technical difficulties with high postoperative complication and mortality rates (14), and, because left tumors are extending proximally, these are often already invading the subaortic space structures (15, 16).

The multidisciplinary assessment is fundamental in order to discuss the indications with the other specialists on a case-by-case basis. Pulmonary function tests, blood gas analysis, and cardiopulmonary exercise tests are mandatories for patient selection in order to exclude patients who could not tolerate a pneumonectomy. Ventilation/perfusion lung test could be performed to estimate the loss of lung function. Preoperative evaluation includes also a CT scan and PET-CT to assess the extent of the tumor above the tracheo-bronchial angle and the possible involvement of surrounding structures. If nodal involvement is suspected, then EndoBronchial Ultrasound (EBUS)/Endoscopic UltraSonography (EUS) or mediastinoscopy is mandatory: surgery is not indicated in two N2 nodal levels above 2R/L or N3 disease (17).

There are only a few clinical series in the literature that differ in the variety of surgical procedures. Intraoperative management is still controversial; the procedure can be performed in a single step or in two steps. The first approach includes left posterolateral thoracotomy, bilateral anterolateral thoracotomy, median sternotomy, clamshell (15, 17, 18), and, more recently, left VATS pneumonectomy followed by right thoracotomy (16). This technique, developed on the wave of an increasing diffusion of the minimally invasive techniques, however, has some disadvantages: it requires a position change, and there are also disadvantages relative to the ventilation during airway anastomosis, which is complicated and difficult to address in an emergency. Two-stage surgery includes a left proximal pneumonectomy, accepting a positive resection margin, followed by a carinal resection from the right side approximately 3 to 5 weeks later (19).

In our center, we have performed a median sternotomy in all cases to provide a good exposure to the carina, avoiding difficulties with the anastomosis behind the aortic arch. In one case, it was necessary to perform a left-sided VATS because of persistent adhesions between the left lower lobe and the thoracic wall with doubtful infiltration of the VI coast. This minimally invasive surgical approach may be useful in severe pleuro-parenchimal adhesions to achieve complete lymphadenectomy and complete hemostasis.

Dissection of the trachea must be limited to the anterior surface and the first 2 cm of the right bronchus, preserving the bronchial irroration as much as possible. The airways are divided and reconstructed in an end-to-end anastomosis before specimen removal (20).

After dissection of the airway, lung resection is performed; access to the hilium can be facilitated by a retractor. In the first two cases, we used a rigid retractor between the superior vena cava and the aortic arch before inserting an octopus-shaped retractor, which causes less trauma to the structures and, at the same time, leading to a less footprint of the surgical field.

Beyond the surgical challenge, we must consider the importance of adequate ventilation and oxygenation during anastomotic reconstruction. Various techniques for intraoperative airway management were presented, such as a single lumen endobronchial tube, cross-field ventilation, high-frequency jet ventilation (HFJV), intermittent apneic ventilation, and extracorporeal membrane oxygenation (21, 22).

A tight collaboration between surgeon and anesthesiologist is fundamental for an optimal airway management. For the first two cases, after the resection of the left lung, the contralateral lung was ventilated through a cross-field ventilation. This technique is associated with impediment in the operative field, a repetitive withdrawal of the endotracheal tube, which leads to a prolonged surgical time and to a risk of injury of the bronchus, as well as lung barotrauma (23). For the second two cases, we decided to change the ventilation mode to the apneic oxygenation technique: before performing the dissection, the patient is hyperventilated and hyperoxigenated with 100% (O_2_) for 10 min in order to obtain an arterial PO_2_ (partial pressure of oxygen) and pCO_2_ (partial pressure of carbon dioxide) levels of 450 or greater and, respectively, 28 mmHg to 35 mmHg. The patient is than in complete apnea, and the airway is then resected. Hyperoxygenation is than obtained throw a small catheter (10F) across the surgical field, connected to a sterile line ensuring O_2_ of 10 L/min to 15 L/min, constantly, associated to a minimal breathing pressure (0–1 mmHg), reducing the footprint of the operative field. Once the anastomosis is complete, the ventilation is assured by the original orotracheal tube (20). The use of a cardiovascular support, like the CPB, has not been used in our technique to minimize the blooding risk due to the circuit heparinization. The challenge in this kind of resections is to ensure both sufficient surgical exposure and adequate ventilation control. Cross-field ventilation with sterile tube can be replaced or partially combined with HFJV or high-flow oxygen insufflation via small-diameter catheters as described. The use of CPB in this type of surgery is considered more of a rescue tool in emergency situations due to the increased risk of bleeding (24). Extracorporeal membrane oxygenation may be indicated if the patient’s condition does not allow safe single-lung ventilation. In this case, it can be effective and allow prolonged apnea avoiding prolonged cross-field ventilation (25).

## 4 Conclusion

Left sleeve pneumonectomy has no standardized approach and, with both intra- and postoperative difficulties, still represents a surgical challenge. With a careful selection of the patients through a collegial discussion and with cooperation between surgical and anesthesiological management, this technique represents a safe procedure with acceptable mortality and morbidity.

## Data availability statement

The original contributions presented in the study are included in the article/**Supplementary Material**. Further inquiries can be directed to the corresponding author.

## Ethics statement

This article does not contain any study with human participants or animals performed by any of the authors. Our institutional review board granted approval and waived the requirement for specific informed consent for this case report. Informed consent was obtained from all subjects involved in the study. Written informed consent has been obtained from the patients to publish this paper. Written informed consent was obtained from the participant/patient(s) for the publication of this case report.

## Author contributions

ST: Conceptualization, Data curation, Supervision, Writing – review & editing, Investigation. DV: Supervision, Writing – review & editing, Validation, Visualization. OS: Supervision, Writing – review & editing, Data curation, Investigation. MT: Data curation, Writing – original draft. LV: Supervision, Validation, Writing – review & editing. AG: Supervision, Validation, Writing – review & editing, Conceptualization, Data curation, Writing – original draft.

## Supplementary material

Supplementary Video 1 Patient #3: *L*eft tracheal sleeve pneumonectomy performed via median sternotomy.

Supplementary Video 2 Pre-operative fibrobronchoscopy of Patient #4 reveals a lesion occupying the left main bronchus, completely blocking the left bronchial tree. Post-operative control was performed 30 days following a left tracheal sleeve lobectomy.

## References

[1] Dartevelle PG Macchiarini P Chapelier AR . Tracheal sleeve pneumonectomy for bronchogenic carcinoma: Report of 55 cases. Ann Thorac Surg. (1988) 46:68–72. doi: 10.1016/s0003-4975(10)65855-9 3382290

[2] Abbott OA . Experiences with the surgical resection of the human carina, tracheal wall, and con-tralateral bronchial wall in cases of right total pneumonectomy. J Thorac Surg. (1950) 19:906–22. doi: 10.1016/s0096-5588(20)31703-7 15415939

[3] Chamberlain JM Mcneill TM Parnassa P Edsall JR . Bronchogenic carcinoma: an aggressive sur-gical attitude. J Thorac Cardiovasc Surg. (1959) 38:727–45. 13809058

[4] Jensik RJ Faber LP Kittle CF Miley RW Thatcher WC El-Baz N . Survival in patients undergoing tracheal sleeve pneumonectomy for bronchogenic carcinoma. J Thorac Cardiovasc Surg. (1982) 84:489–96. doi: 10.1016/s0022-5223(19)38976-7 7121039

[5] Dartevelle P Macchiarini P . Carinal resection for bronchogenic cancer. Semin Thorac Cardiovasc Surg. (1996) 8:414–25. 8899929

[6] Regnard J-F Perrotin C Giovannetti R Schussler O Petino A Spaggiari L . Resection for tumors with CARINAL involvement: Technical aspects, re-sults, and prognostic factors. Ann Thorac Surg. (2005) 80:1841–6. doi: 10.1016/j.athoracsur.2005.04.032 16242466

[7] Grillo HC . Tracheal surgery. Scandinavian J Thorac Cardiovasc Surg. (1983) 17:67–77. doi: 10.3109/14017438309102383 6306761

[8] Mathisen DJ Grillo. HC . Carinal resection for bronchogenic carcinoma. J Thorac Cardiovasc Surg. (1991) 102:16–23. doi: 10.1016/s0022-5223(19)36579-1 2072721

[9] Grillo HC . Carcinoma of the lung: What can be done if the Carina is involved? Am J Surg. (1982) 143:694–6. doi: 10.1016/0002-9610(82)90038-1 6283925

[10] Roviaro G Varoli F Rebuffat C Scalambra SM Vergani C Sibilla E . Tracheal sleeve pneumonectomy for bronchogenic carcinoma. J Thorac Cardiovasc Surg. (1995) 110:567–8. doi: 10.1016/s0022-5223(95)70267-9 7637386

[11] Mitchell JD Mathisen DJ Wright CD Wain JC Donahue DM Moncure AC . Clinical experience with Carinal resection. J Thorac Cardiovasc Surg. (1999) 117:39–53. doi: 10.1016/s0022-5223(99)70468-x 9869757

[12] Mitchell JD Mathisen DJ Wright CD Wain JC Donahue DM Allan JS . Resection for bronchogenic carcinoma involving the carina: Long-term results and effect of nodal status on outcome. J Thorac Cardiovasc Surg. (2001) 121:465–71. doi: 10.1067/mtc.2001.112832 11241081

[13] Eichhorn F Storz K Hoffmann H Muley T Dienemann H . Sleeve pneumonectomy for Central Non-Small cell lung cancer: Indications, complications, and survival. Ann Thorac Surg. (2013) 96(1):253–8. doi: 10.1016/j.athoracsur.2013.03.065 23673074

[14] Roviaro G Varoli F Romanelli A Vergani C Maciocco M . Complications of tracheal sleeve pneu-monectomy: Personal experience and overview of the literature. J Thorac Cardio-vascular Surg. (2001) 121(2):234–40. doi: 10.1067/mtc.2001.111970 11174728

[15] Aigner C Lang G Klepetko W . Sleeve pneumonectomy. Semin Thorac Cardiovasc Surg. (2006) 18(2):109–13. doi: 10.1053/j.semtcvs.2006.05.005 17157229

[16] Fujino T Tanahashi M Yukiue H Suzuki E Yoshii N Shitara M . A new approach to left sleeve pneumonectomy: Complete vats left pneumonectomy followed by right thoracotomy for Carinal resection and Reconstruction. Surg Case Rep. (2018) 4(1):91. doi: 10.1186/s40792-018-0496-2 30097740 PMC6086918

[17] Macchiarini P Altmayer M Go T Walles T Schulze K Wildfang I . Technical innovations of carinal resection for nonsmall-cell lung cancer. Ann Thorac Surg. (2006) 82(6):1989–97. doi: 10.1016/j.athoracsur.2006.07.016 17126096

[18] Maeda M Nakamoto K Tsubota N Okada T Katsura H . Operative approaches for left-sided carinoplasty. Ann Thorac Surg. (1993) 56(3):441–6. doi: 10.1016/0003-4975(93)90877-k 8379714

[19] Deslauriers J Grégoire J Jacques LF Piraux M . Sleeve pneumonectomy. Thorac Surg Clinics. (2004) 14(2):183–90. doi: 10.1016/s1547-4127(04)00012-x 15382294

[20] Gonfiotti A Jaus MO Barale D Macchiarini P . Carinal resection. Thorac Surg Clinics. (2014) 24(4):477–84. doi: 10.1016/j.thorsurg.2014.08.001 25441142

[21] Gritsiuta AI Bakhos CT Abbas AE Petrov RV . High-frequency jet ventilation jets the way to minimally invasive carinal re-section? J Thorac Dis. (2022) 14(12):4590–2. doi: 10.21037/jtd-22-1196 PMC9840025 36647480

[22] Rea F Marulli G Schiavon M Zuin A Hamad AM Feltracco P . Tracheal sleeve pneumonectomy for Non Small Cell Lung Cancer (NSCLC): Short and long-term results in a single institution. Lung Cancer. (2008) 61(2):202–8. doi: 10.1016/j.lungcan.2007.12.015 18280612

[23] Bellier J Sage E Gonin F Longchampt E Chapelier A . Radical carinal resection for a glomic tumor. Ann Thorac Surg. (2016) 102(2):e143-5. doi: 10.1016/j.athoracsur.2016.01.023 27449451

[24] Lang G Ghanim B Hötzenecker K Klikovits T Matilla JR Aigner C . Extracorporeal membrane oxygenation support for complex tracheo-bronchial procedures†. Eur J Cardio-Thoracic Surg. (2014) 47(2):250–6. doi: 10.1093/ejcts/ezu162 24740936

[25] Voltolini L Salvicchi A Cianchi G Bongiolatti S . Extracorporeal membrane oxygenation support in carinal resection for recurrent chondrosarcoma after previous distal tracheal resection. Interactive Cardiovasc Thorac Surg. (2022) 35(1):ivac148. doi: 10.1093/icvts/ivac148 PMC9216038 35652753

